# Natural orifice transluminal endoscopic surgery: Past, present and future

**DOI:** 10.4103/0972-9941.33271

**Published:** 2007

**Authors:** Jonathan P Pearl, Jeffrey L Ponsky

**Affiliations:** Department of Surgery, Case Western Reserve University School of Medicine, Cleveland, OH, USA

Natural orifice transluminal endoscopic surgery (NOTES) first gained notoriety with the initial laboratory report of transgastric peritoneoscopy in 2004.[1] Since then the medical community and the general public has been captivated with the idea of “no-scar” abdominal surgery. In animal models, translumenal organ resections have been shown to be feasible[2–7] and an unpublished clinical case series of transgastric appendectomies has been discussed across the world. However, as data accumulate we are learning that NOTES techniques require substantial refinement before achieving clinical applicability.

## THE BASICS

Natural orifice transluminal endoscopic surgery (NOTES) involves accessing the abdominal cavity via one of the bodies' natural orifices (mouth, anus, vagina or urethra). A flexible endoscope is advanced into the peritoneal cavity after puncturing one of the viscera (stomach, colon, vagina or bladder). Endoscopic insufflation creates a pneumoperitoneum and the appropriate working space. Conventional endoscopic instruments are advanced through the working channels of the endoscope in order to perform the operation.

That simple action of purposely puncturing one of the viscera has raised many questions: What are the infectious implications? How can reliable closure be achieved? Is it practical to make a viscerotomy in the era of minimal access laparoscopic surgery? These questions all require cogent answers before proceeding to clinical (NOTES). Eliminate natural orifice transluminal endoscopic surgery.

## SOME ADVANTAGES

There are many potential advantages of NOTES over conventional surgery. Much like laparoscopy has demonstrated less physiologic impact than laparotomy, NOTES may cause less physiologic insult than either laparoscopy or laparotomy. Laboratory studies are underway investigating cytokine levels with NOTES in comparison to conventional operations. Furthermore, natural orifice surgery may negate the possibility of wound complications and reduce the formation of intraabdominal adhesions.

Given the portability of NOTES equipment, natural orifice surgery is suited for an intensive care unit. Moving the equipment to the patient, rather than vice versa, might reduce the resource requirements and potential complications of transporting a patient to the operating room. Moreover, NOTES could be performed under conscious sedation, rather than general anesthesia, again favoring intensive care unit (ICU)-based procedures.

Lastly are the cosmetic benefits of NOTES. While NOTES would offer abdominal operations without skin incision, this fact should not be the driving force behind NOTES. The public at large may be enchanted with no-scar surgery, but physicians and surgeons should make rational decisions based on sound data.

## NUMEROUS DRAWBACKS

Besides the fact that intentionally puncturing one of the viscera contravenes surgical dogma, there are many shortcomings of NOTES in its current state. It has become plainly obvious that current instrumentation is inadequate to perform NOTES. Making the viscerotomy and accessing the abdomen is feasible using standard endoscopic equipment, but beyond those steps, technical advances are imperative for the success of NOTES.

A stable operating platform is necessary to safely conduct precise abdominal operations. The flexibility of the endoscope provides the ability to maneuver to the organ of interest. However, the flexibility also precludes stabilization of the tip of the scope. A stiffening overtube might solve this problem.

One of the basic tenets of laparoscopy is triangulation of optics and instrumentation. The current version of end-viewing endoscopes precludes such triangulation in natural orifice surgery. The working channels are in line with the optical view, limiting range of motion of instruments and obscuring the view of the operative field.

The published NOTES feasibility studies have used standard endoscopic instrumentation to perform abdominal operations. In clinical practice biopsy forceps and snares may be adequate for simple manipulations, but complex surgery, such as that on the biliary tree, demands improved instruments. Again, a triangulating endoscope would permit concomitant retraction and dissection of tissue. Innovative instruments might allow greater degrees of freedom than are currently available with conventional endoscopic tools.

Foremost is the requirement for a reliable method for closure of the viscerotomy. In a porcine model, some groups have advocated no closure of a gastrotomy. Others have successfully employed endoscopic clips for closure. Neither of these comports with the surgical principle of full thickness closure of a viscerotomy. Endoscopic clips and some endoscopic sewing machines only approximate the mucosa, which contravenes surgical dogma. Therefore, technical advances are necessary to develop a method of reliable full-thickness, watertight visceral closure.

## THE WAY FORWARD

Members of the American Society for Gastrointestinal Endoscopy and the Society of American Gastrointestinal and Endoscopic Surgeons joined to form a new group, Natural Orifice Surgery Consortium for Advancement and Research (NOSCAR). The purpose of forming NOSCAR was to regulate the progress toward clinical NOTES and to ensure the safety of future procedures.

NOSCAR leadership. Eliminate recently published its recommendations for the progression of NOTES in the “White Paper”.[8] A clarion call for rigorous laboratory research was sounded. Investigating the immunologic and infectious implications of NOTES was emphasized. Moreover, NOSCAR recommended that all teams investigating NOTES cases be conducted under the guidance of local Institutional Review Boards and entered into a centralized database for tracking of outcomes.

Perhaps most importantly was the exhortation for cooperation between gastroenterologists and surgeons in advancing NOTES. NOSCAR recommended that all teams investigating NOTES be comprised of at least one surgeon and one gastroenterologist working in cooperation to advance the fledgling field of NOTES.

## QUALIFICATIONS

Should clinical NOTES come to fruition, the issue of qualifications and credentialing for performing translumenal surgery arises. Should gastroenterologists or surgeons be the practitioners of NOTES?

In all likelihood, only a small fraction of highly trained gastroenterologists and surgeons will become NOTES surgeons. Performing NOTES is contingent upon demonstrating expertise in flexible endoscopy, abdominal anatomy, surgical principles and managing complications. The initial expertise will be acquired through diligent laboratory work followed by judicious clinical use.

Today's principal investigators of laboratory and clinical NOTES will become tomorrow's instructors in NOTES. Subsequently a new fellowship in endoscopic surgery might arise. A year of training in endoscopic surgery could follow a fellowship in advanced endoscopy. It would be open to both gastroenterologists and surgeons with an interest in acquiring the necessary skills and knowledge base to perform transgastric surgery.

## BRIDGE PROCEDURES

In addition to sound laboratory research, a stepwise clinical approach to clinical NOTES allows safe progress. Transgastric peritoneoscopy under laparoscopic guidance serves as a bridge to NOTES, assessing the practicality and safety of transgastric surgery in humans. These bridge procedures might be performed in patients requiring laparoscopic operations requiring a gastrotomy. For example, posterior gastric wall GI stromal tumors might be resected through an anterior gastrotomy. After making the gastrotomy the endoscope could be passed into the abdominal cavity and peritoneoscopy could be performed with laparoscopic supervision.

Bridge procedures permit investigation of some of the critical issues of NOTES: Infectious implications and scope positioning. In some reports using a porcine model there was a high rate of intraabdominal abscess formation after transgastric peritoneoscopy. Whether this was a function of the porcine model or the result of transgastric endoscopy could be answered by clinical bridge procedures. Moreover, the maneuvers necessary to visualize the various abdominal organs could be assessed, thereby providing insight into the placement of the viscerotomy and the necessary technical refinements.

## FUTURE APPLICATIONS

The initial applications of NOTES will likely transcend the boundaries of conventional general surgery. There is little morbidity in performing a laparoscopic cholecystectomy, for example and intentionally making gastrotomy for a cholecystectomy may not be sage. However, transgastric abdominal operations in intensive care unit patients may offer many benefits.

The portability of NOTES equipment is ideal for performing transgastric surgery in the intensive care unit. A large percentage of ICU patients rely on temporary respiratory support, but weaning from the ventilator might be facilitated by diaphragm pacing. Transgastric placement of diaphragm pacing leads has several potential benefits over laparoscopic placement.[9] The resource requirements and hazards of transportation would be negated and the procedure could be performed under sedation at the bedside.

Another potential ICU application is transgastric abdominal exploration for compromised bowel in suspected cases of mesenteric ischemia. Detecting necrosis of the entire small bowel would obviate a trip to the operating room for a nontherapeutic laparotomy. Findings of limited ischemia or necrosis would serve to select those patients who might benefit from a laparotomy.

Managing complications of percutaneous endoscopic gastrostomy tubes could be performed endoscopically in the ICU. We have recently performed a NOTES transgastric “rescue” of a dislodged PEG in the ICU under conscious sedation.[10] An elderly neurologically-compromised patient removed his PEG 3 days after initial placement. A contrast study through the previous PEG site revealed free intraperitoneal dissemination of contrast. In order to spare the patient a laparotomy a flexible endoscope was advanced through the previous gastrotomy into the peritoneal cavity. A percutaneously placed wire was snared with the endoscope and the PEG was restored in its previous position. The patient recovered uneventfully without evidence of intraabdominal infection.

## CONCLUSIONS

We may be a long way from routine clinical applications of NOTES, but we are making steady progress. Circumspect laboratory investigation is critical to understanding the physiologic impact of translumenal surgery. Moreover, all clinical NOTES should be performed under the aegis of a cogent clinical trial. Finally, a spate of new instrumentation is vital for performing safe NOTES.

There may be a time when transgastric cholecystectomy is routine, but that time is not now. Patient safety, not the advancement of NOTES, is paramount. Presently, we should perform diligent laboratory research and possibly some limited clinical trials, awaiting the data confirming our suspicion that NOTES is the way of the future.
